# Assessment of water use efficiency in arid ecosystems of the coastal land of Egypt using MODIS dataset

**DOI:** 10.1038/s41598-026-67232-3

**Published:** 2026-09-05

**Authors:** Amira M. Hotaiba, Boshra B. Salem, Marwa Waseem A. Halmy

**Affiliations:** Department of Environmental Sciences, Faculty of Science, Alexandria University, Moharm Bek, P.O. Box: 21511, Alexandria, Egypt

**Keywords:** Carbon–water interactions, Anthropogenic impacts, Land use, Evapotranspiration, GPP, Biogeochemical cycles, Ecology, Ecology, Environmental sciences, Hydrology

## Abstract

In drylands, the limited availability of reliable measurements makes it more difficult to understand how climatic and biogeochemical factors shape ecosystem processes across spatial gradients compared to mesic or humid regions. Water use efficiency (WUE) is a key physiological indicator for assessing interactions between carbon and water cycles in any ecosystem. Yet, in dryland ecosystems with sparse vegetation where evaporation represents a large fraction of total evapotranspiration (ET), the response of WUE to land use changes needs more investigation. To address this gap, the present study examines the spatial and temporal patterns of WUE in coastal dryland ecosystems of Egypt to better understand how land use practices affect water dynamics. Using an integrated approach that combines remote sensing products, WUE trends were assessed across representative sites. The results show high spatial variation in the WUE pattern over the 25-year period, with absolute values ranging from below 1.0 × 10^−3^ g C mm^−1^ in sparsely vegetated zones up to 2.9 × 10^−3^ g C mm^−1^ in reclaimed lands. The highest values of WUE are for the vegetated areas of croplands and reed vegetation, where the biomass reflects the growth of developed vegetation. The natural land covers are subjected to various changes due to human activities and expansion in the reclaimed agricultural areas where the urbanization activities increase ET and reduce WUE. These insights can contribute to a better understanding of dryland ecosystem functioning and can inform sustainable water and land management strategies in arid regions facing increasing water scarcity.

**Supplementary Information:**

The online version contains supplementary material available at https://doi.org/10.1038/s41598-026-67232-3.

## Introduction

Arid and semi-arid ecosystems are among the most vulnerable regions to the impacts of climate change^1–3^. Characterized by sparse vegetation, erratic rainfall, and high temperatures, these regions are especially susceptible to shifts in climate patterns and related ecological stresses^4^. Drylands can act as a sink for carbon if it was managed under conservation strategies with adequate policies, therefore it is important to measure how drylands contribute to the water usage in any ecosystems^5–7^.

Egypt encompasses diverse ecosystems, each with unique flora and fauna across various agroecological zones: the North Coastal Belts, Nile Valley, Inland Sinai and Eastern Desert, and the Western Desert, each facing unique agricultural and environmental challenges^8^. Egypt’s ecological regions are abundant and varied, encompassing the fertile Nile Valley and the expansive deserts that dominate much of the country’s territory^9,10^. One of the most pressing challenges facing these ecosystems is the scarcity of water resources. With limited rainfall and vast desert areas, Egypt relies heavily on the Nile River, which has led to the development of unique and fragile environmental conditions, particularly in the Nile Delta region^11^. Notably, over 90% of Egypt’s land comprises arid and semi-arid habitats, which incorporate diverse ecosystems such as wetlands, deserts, and marine and freshwater habitats^12^. However, due to the limited availability of reliable measurements in drylands, understanding how climatic and biogeochemical factors influence ecosystem processes across different spatial gradients remains more challenging than in mesic or humid regions^13^.

Water use efficiency (WUE), the ratio of carbon gain to water loss, is an important physiological indicator in assessing the interactions between the carbon and water cycles in any ecosystem^14^. WUE is regarded as an important characteristic of ecosystem function that links between carbon gain (i.e., photosynthesis) and water loss (i.e., transpiration) in plants or terrestrial ecosystems representing the interactions between carbon sequestration and water consumption^14–16^.

At the ecosystem scale, WUE is typically defined as the ratio of gross primary productivity (GPP) or net ecosystem productivity (NEP) to evapotranspiration (ET)^17^. Understanding the mechanisms of WUE patterns and variations at different scale levels is of great importance to the projections of global water and carbon balance under the changing climate^18^.

The pattern of dryland ecosystems’ WUE and the responses to climate variability and temperature change are not well understood. In these ecosystems evaporation may account for a sizeable proportion of the entire ecosystem ET due to the sparse vegetation^19^. Water availability is critical to the spatial variability of carbon fluxes in arid regions. Therefore, quantifying WUE for both natural and managed ecosystems in dryland regions is fundamentally important for accurately predicting the water balances and informing the sustainable management of water resource in these ecologically fragile areas^20,21^. Human activities, such as irrigation and agricultural practices, have significantly altered water distribution and usage strategies, which in turn have affected the magnitude and patterns of carbon and water fluxes in dryland ecosystems^22,23^. Patterns of WUE in drylands regions are difficult to investigate due to low data values and limited biomass productivity^21^.

Satellite-based remote sensing is a powerful tool for assessing water use efficiency (WUE) across ecosystems at regional to global scales. By integrating vegetation indices, such as the Normalized Difference Vegetation Index (NDVI), with satellite-derived estimates of gross primary production (GPP) and evapotranspiration (ET), quantifying large-scale patterns of WUE with consistent spatial and temporal coverage is possible^24,25^. Tower-based WUE measurements serve as a benchmark for testing the reliability and accuracy of satellite-derived global WUE estimates^26^. Sensors like NASA’s MODIS onboard the TERRA and AQUA platforms provide long-term datasets that enable the detection of spatial heterogeneity, temporal variability, and responses of WUE to environmental drivers, including droughts and heatwaves^27,28^. Compared to flux towers ground-based measurements, which provide localized and high-precision observations, remote sensing offers broader spatial coverage and scalability^29,30^. This integrated approach enhances our understanding of ecosystem productivity and water cycling, in addition to supporting the development of predictive models evaluating climate change impacts on terrestrial carbon–water interactions^15,16,31^.

Measuring WUE and studying its pattern for any ecosystem is important as it provides valuable insights by: (1) capturing the spatiotemporal variations of water availability, thereby improving the accuracy of terrestrial water and carbon cycle simulations^32^, (2) assessing drought impacts on productivity through biomass accumulation^33^, (3) evaluating ecosystem resilience to climate variability under rapidly changing conditions^18,34^, (4) detecting early signs of environmental degradation and ecosystem disturbance^15,16^, and (5) monitoring vegetation dynamics as an indicator of ecosystem health and biodiversity^35^. The study by Alsafadi^36^ indicated that fluctuations in water-use efficiency (WUE) in arid ecosystems are primarily driven by evaporation, whereas in semi-arid and sub-humid environments, WUE variability is largely governed by biological processes, including changes in stomatal conductance, leaf area dynamics, photosynthetic capacity, and vegetation phenology, which together regulate the balance between carbon assimilation and transpiration^15,16,37–39^. Further studies are needed to investigate how human activities affect the redistribution of water and how possible that influence water availability and the WUE the across different dryland ecosystems^14^.

This study represents the initial attempt to assess the water use efficiency (WUE) of arid coastal ecosystems in Egypt using remotely sensed data. The study endeavors to evaluate how carbon and water exchanges are regulated under water-limited conditions, while emphasizing the critical need for high-resolution satellite data on gross primary productivity (GPP) and evapotranspiration (ET). More specifically the study sought to: (1) analyze the spatial and temporal patterns of water use efficiency (WUE) in coastal dryland ecosystems of Egypt over the last 25 years; (2) identify key drivers of variation in WUE and their implications for ecosystem functioning under water-limited conditions, in particular role of climatic variability and land use practices on coupled water and carbon dynamics; and (3) provide insights that support sustainable water and land management strategies in arid regions under increasing water scarcity.

## Materials and methods

### Study area

The Mediterranean coastal zone of Egypt extends for more than 1200 km and falls within the dry arid climatic region with annual mean temperature ranging from 15.1 °C to 25.3 °C^40^. The area encompasses diverse land cover types, including brackish coastal wetlands, agricultural lands concentrated in the deltaic section, newly reclaimed lands west of the delta, coastal dunes, salt marshes, and natural sand and gravel flats in the desert interior^41,42^.

The northwestern coastal zone of Egypt extends from Alexandria to El-Salloum, covering approximately 550 km, and is characterized by diverse geomorphological features. This section of the coast is marked by a chain of strikingly white calcareous granular sand dunes^12^. South of the dune belt, two to three ridges run parallel to the Mediterranean shoreline, composed mainly of sand and shell debris^43^. The region also includes saline depressions with calcareous-sandy soils as well as non-saline depressions, which are common in valleys and plains of the western sector^44,45^.

The eastern Mediterranean coast encompasses the North Sinai shoreline and areas adjacent to the Nile Delta beaches. This region is dominated by sabkhas, coastal flats, and sand sheets^46^. The northern two-thirds of the Sinai Peninsula consists of a limestone plateau rising from the Mediterranean and extending southwards, interspersed with wide plains of sand dunes reaching 80–100 m in height^12,47^.

**Fig. 1:**
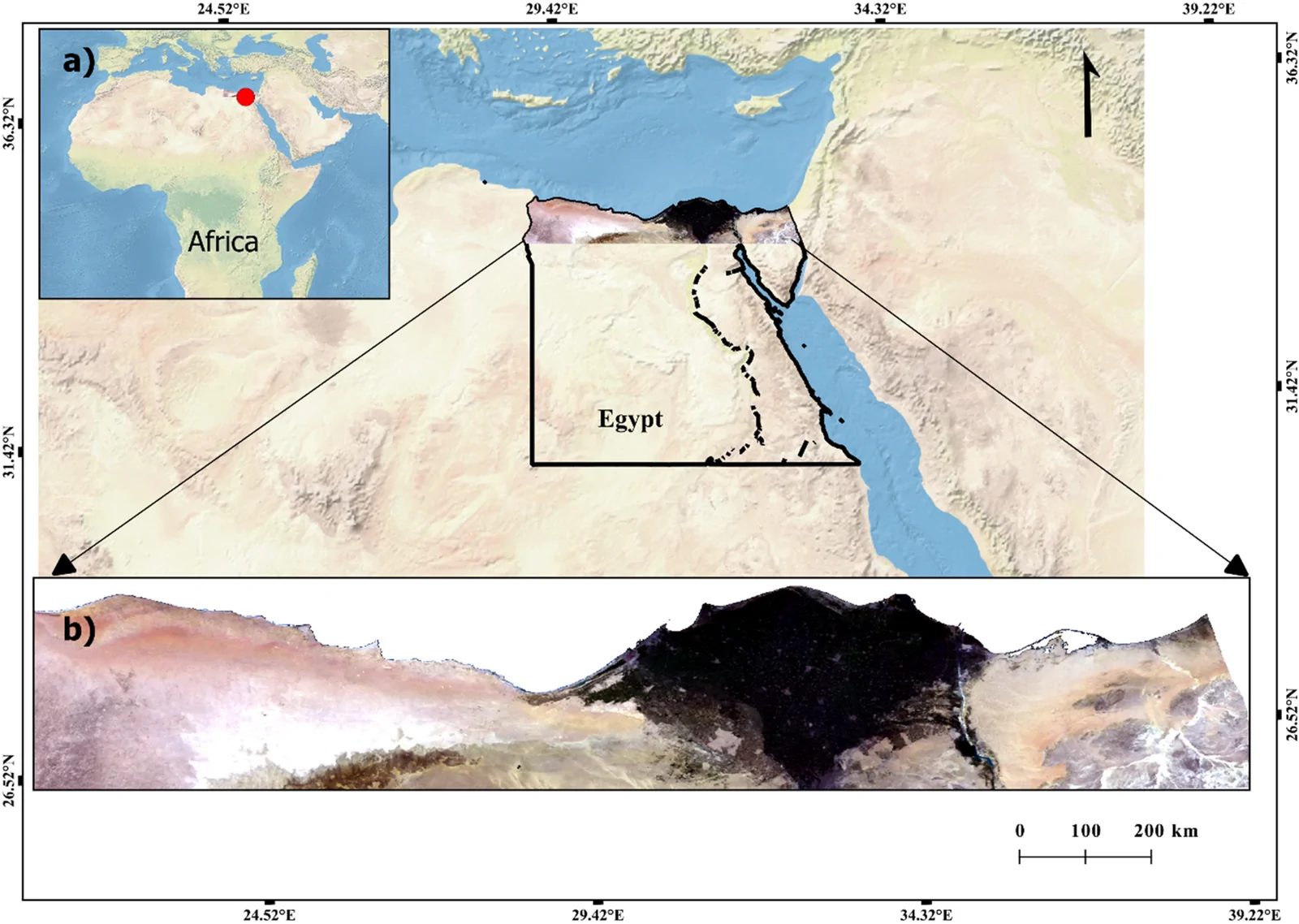
(**a**) Location of the study area in Egypt. The inset map highlights Egypt’s position within North Africa and the Middle East (red dot), (**b**) MODIS scene showing the study area, the data represent the surface reflectance image at 500 m resolution displayed as a true-colour composite (bands 1, 3, and 4). *Source*: Terra MOD09A1 Version 6.1 product^51^. Map generated by the authors using QGIS Desktop version 3.34.8^52^ https://qgis.org. The base map raster data was sourced from Natural Earth map dataset (http://www.naturalearthdata.com/), which is in the public domain and freely available for all uses. Map licence: CC BY.

In recent decades, the Mediterranean coastal lands of Egypt have undergone rapid transformations driven by urban expansion associated with population growth, as well as large-scale agricultural reclamation projects, particularly west of the Nile Delta^48,49^. These areas are ecologically fragile and highly dynamic, making them vulnerable to environmental pressures and human-induced change^50^. Figure 1. shows the study area and its geographic context.

### Remote sensing dataset

The Moderate Resolution Imaging Spectroradiometer (MODIS) dataset collection of Gross Primary Productivity (GPP) is MOD17A2H Version 6.1^53^, which is a cumulative 8-day composite of values with 500-m pixel size based on the radiation use efficiency concept that can be potentially used as inputs to data models to calculate terrestrial energy, carbon, water cycle processes, and biogeochemistry of vegetation. The Evapotranspiration dataset was acquired from MODIS/Terra Net Evapotranspiration Gap-Filled 8-Day L4 Global (MOD16A2GF) Version 6.1 produced at 500-m pixel resolution^54^. The Evapotranspiration/Latent Heat Flux (ET/LE) product is a year-end gap-filled 8-day composite dataset featuring full-year temporal gap-filling to account for missing data due to cloud cover or poor atmospheric conditions. The algorithm used in this product collection is based on the logic of the Penman–Monteith equation, which includes inputs of daily meteorological reanalysis data along with MODIS remotely sensed data products such as vegetation dynamics, albedo, and land cover^54^. Google earth engine (GEE) platform^55^ was used for acquisition and processing of the dataset. According to the availability of the dataset, the time range of the study was from 2000 to 2024, which is a 25-years period. All the datasets were clipped to the specified area of interest (AOI) which represents the north coastal area of Egypt. The data sets used in the study are illustrated in Table 1.

**Table 1:** Summary of the data used in this study.

Data	Source	Spatial resolution	Temporal resolution	Unit
Satellite images	MOD09A1.061 Terra Surface Reflectance 8-Day Global 500m Vermote^51^	500 m	8 days	Surface spectral reflectance
Gross primary productivity (GPP)	MOD17A2H Terra Gross Primary Productivity 8-Day Global 500m Running^53^	500 m	8 days	kg-Carbon/m^2^
Evapotranspiration (ET)	MOD16A2GF Terra Net Evapotranspiration Gap-Filled 8-Day Global 500m Running^54^	500 m	8 days	kg/m^2^/8 day

### The classification process

The dataset used for LULC classification was the MODIS Terra (MOD09A1 Version 6.1) surface reflectance product at 500 m spatial resolution^51^. This dataset provides atmospherically corrected reflectance in 7 spectral bands, with values selected from the best observations within an 8-day composite period. Data for the study area, covering the Mediterranean coastal zone of Egypt, were filtered for the period July–September to minimize cloud cover and ensure stable surface conditions. QA bands were applied to mask clouds and low-quality pixels. All data were processed and analyzed within the Google Earth Engine (GEE) platform.

The classification process consisted of three stages: (1) unsupervised classification to explore spectral separability and guide sample allocation, (2) supervised classification using the Random Forest (RF) algorithm^56^, and (3) accuracy assessment. For the supervised classification step, training and validation samples were derived from a combination of high-resolution Google Earth imagery and existing land cover maps. A stratified random sampling strategy was applied, and the samples were split into 70% for training and 30% for validation.

The final LULC classes included in the classification: urban/built-up, agricultural land, wetlands, water bodies, coastal dunes, natural vegetation, barren land, and desert flats. To improve class separability, ancillary data were included with the MODIS spectral reflectance bands. Ancillary data included two spectral indices, the Normalized Difference Vegetation Index (NDVI)^57^, and the Normalized Difference Built-up Index (NDBI)^58^. In addition, topographic variables were obtained from the Shuttle Radar Topography Mission (SRTM DEM, 30 m)^59^, including elevation, slope, and aspect. Two proximity metrics were also included: distance to water bodies, and distance to coast. A wetland-derived water mask was converted into a raster layer and used to calculate distance-to-water surfaces, while the Open Shoreline dataset^60^ was used to compute distance-to-coastline metrics. Soil data including the soil organic carbon stock (SOCS) derived from SoilGrids^61^ were also integrated. These variables were used alongside MODIS reflectance bands, resulting in a total of 15 predictor variables employed in the classification process. Their integration ensured high accuracy and improved separation of overlapping classes.

The selection of the training sites was conducted carefully to represent the different LULC existing within the area of study. The process was guided by field observations, maps, and Google Earth; other previous studies and literature were also utilized for assistance in the classification process. The RF classifier was configured with 500 trees, and out-of-bag error estimation for internal validation. The RF classifier is a machine learning algorithm that can be used for land cover classification (e.g.,^62–65^). It consists of an ensemble of tree-structured classifiers^56^ and consistently demonstrated better accuracy compared to other classifiers^66^. The key advantage of RF classifier is its insensitivity to noise or overtraining, superiority to many tree-based algorithms, and suitability for handling unbalanced and heterogenous data sets^67,68^.

For classification accuracy assessment the Train/Test split methodology on GEE was initially used. To provide a more robust assessment of the classification accuracy, the land-cover maps were evaluated using an expanded independent validation dataset comprising additional reference points distributed across all land cover categories. The land-cover classification itself was not modified following the initial analysis; the training data, classification algorithm, predictor variables, and model parameters remained unchanged. The expanded validation dataset was used solely to obtain a more representative estimate of classification accuracy by increasing the number and spatial distribution of validation samples, particularly for underrepresented classes. Accuracy evaluation was conducted using a confusion matrix, from which overall accuracy, producer’s accuracy, user’s accuracy, and the Kappa statistic were derived following standard accuracy assessment procedures^69^. A total of 1,500 validation points were generated and allocated proportionally among land-cover classes. These points were visually interpreted using high-resolution imagery available in Google Earth and subsequently compared with the classified land-cover map within Google Earth Engine to construct the confusion matrix.

### Water use efficiency (WUE)

Calculation of WUE was conducted to assess the change in the carbon gain to water loss through Evapotranspiration (ET) in the ecosystems. WUE was estimated based on the ecosystem variables of GPP and ET as following:

1 $$WUE = \frac{GPP}{{ET}}$$

Where GPP is annual gross primary productivity measured in kg Carbon/m^2^, and *ET* is evapotranspiration measured in kg/m^2^/8day from 2000 to 2024 covering 25 years period. Because both MOD17A2H and MOD16A2GF products share the same 8-day temporal grid and sinusoidal tile system, the data layers are inherently synchronized. The raw MODIS ET unit (kg/${\mathrm{m}}^{2}$) was converted to millimeters, assuming a water density of 1000 kg ${\mathrm{m}}^{-3}$, where 1 kg/${\mathrm{m}}^{2}$ of water equates to 1 mm of depth. To ensure standard ecological reporting, the resulting values were converted from (kg C) to (g C). The final WUE is expressed in g C ${\mathrm{m}\mathrm{m}}^{-1}$.

Calculation of WUE for the study area was conducted using the Google Earth Engine (GEE) platform, which enabled efficient processing of large-scale satellite datasets. The MODIS dataset products of ET and GPP are available as 8-day composite products over each year. The annual sum of both GPP and ET was calculated and the ratio of the WUE was measured according to the above-mentioned equation. To depict the spatial patterns of WUE, the assessment of WUE values across the different land cover classes was conducted using the zonal statistics tool in ArcGIS 10.2^70^. The zonal statistics tool was used to calculate the mean and standard deviation of values within each land cover class. Temporal trends for each class were then evaluated using the Mann–Kendall test to determine the significance of observed changes.

### Trend analysis

To evaluate the trend of long-term changes in WUE during the 25-years period, the Modified Mann–Kendall (MK) trend test^71^ was employed on the results. The Modified Mann–Kendall test accounts for autocorrelation that often exists in the hydro-climatic time series by correcting the variance for the MK statistic (*S*), based on the autocorrelation structure of the series.

The magnitude of the trend was quantified using the Sen’s Slope estimator, this estimator is robust against outliers and missing values, which calculates the median of the slopes of all pairs of data points. It is particularly suitable for satellite-derived GPP and ET datasets in arid and semi-arid regions. All statistical analyses were performed at a 95% confidence in R^72^.

### LUCC detection and analysis

Post-classification land use/cover change detection (LUCC) compares independently classified remote sensing images from different time periods to identify land use and land cover changes^73,74^. Among change detection techniques, post-classification LUCC is considered more accurate and provides detailed “from–to” change information^74^, which most other methods cannot offer. However, the reliability of this approach depends on the accuracy of the classifications, as errors during classification and preprocessing can significantly affect the results^75–77^. The overall error in the change map reflects the combined classification errors of the images used^73,78^. Despite these challenges, the method minimizes issues caused by varying atmospheric conditions between scenes and allows the use of images from different sensors^73^. In this study, the LULC maps generated earlier were used to quantify and analyze spatial and temporal changes across the different land cover classes. Land cover change analysis was conducted using both absolute and percentage change metric. The absolute change represents the difference in area (km^2^) for each land cover class between the year 2000 and 2024:

$${\text {Absolute\;change}}\left({\mathrm{km}}^{2}\right)= {\mathrm{Area}}_{2024}- {\mathrm{Area}}_{2000}$$

The percentage change for each land cover class expresses the difference in area relative to the baseline year (2000), calculated as:

$${\text {Percentage\;change}} \left(\mathrm{\%}\right)= \left(\frac{\text {Absolute\;change}}{{\mathrm{Area}}_{2000}}\right)\times 100$$

A positive value indicates increase in the land cover class, while a negative value reflects a decrease.

To explore relationships between land cover dynamics and water use efficiency (WUE), the Pearson correlation coefficient^79^. was estimated. This statistical measure quantifies the strength and direction of the association between two continuous variables, ranging from −1 to + 1. A value of −1 indicates a strong negative correlation (one variable increases the other decreases), + 1 indicates a strong positive correlation (both variables are increasing or decreasing together), and values near zero suggest weak association.

A correlogram matrix was generated to depict the pattern of association in the year-to-year changes in WUE across different land cover categories based on the Pearson correlation results. The analysis was conducted within the framework of R 4.3.3^80^.

### Assessment of the landscape conservation state (ILC)

To assess the conservation state of the landscape, the Index of Landscape Conservation State (ILC) was quantified following the hierarchical framework proposed by Pizzolotto & Brandmayr^81^ and Capotorti et al.^82^. The ILC index measures the structural and ecological condition of the landscape where high values indicate areas dominated by natural habitats with minimal disturbance, while low values reveal heavily transformed landscapes.

An Ecological Quality Class (EQC) was assigned to each land cover category. The classes evaluate the degree of naturalness or anthropogenic pressure and were grouped into two categories: (1) Man-made landscape, including urban areas, agricultural lands, and reclaimed land, and (2) Natural habitat, including coastal wetlands, salt marshes, reed vegetation, bare sand plains, and rocky surfaces. ILC is derived using a moving-window approach that measures the local composition of EQC levels surrounding each pixel. Within each window, the total number of natural pixels (N_nat_) and man-made pixels (N_man_) was computed using binary masking and convolution operators in GEE.

The ILC was then calculated using the normalized difference formula where *n* = number of EQC classes (n = 4). The Index of Landscape Conservation (ILC) was computed as:

$$ILC = \frac{{N}_{nat} - {N}_{man}}{{N}_{nat}+ {N}_{man}}$$

The result of the index ranges from − 1 to + 1, where; (− 1) is a fully man-made landscape, (0) is an equally mixed or highly fragmented landscape, and (+ 1) is a fully natural landscape.

The change detection between the ILC index for each year was produced to compare the difference in land use from the initial time of 2000 to the end time of 2024 as follows:

$$\Delta {\mathrm{ILC}}\, = \,{\mathrm{ILC}}\_{2}0{24}{-}{\mathrm{ILC}}\_{2}000$$

To facilitate ecological interpretation, the produced change detection map was categorized into five landscape degradation categories based on the range of ΔILC values according to Capotorti et al.^82^. These categories served as the final output map for landscape conservation change. All maps presented in the study were produced using ArcGIS 10.2^70^ and 10.3^83^ (Table 2).

**Table 2:** Landscape degradation categories based on the classification of the index of landscape conservation (ILC) values.

Class	Degree	Category
1	ΔILC < −0.3	Strong degradation
2	−0.3 ≤ ΔILC < −0.1	Moderate degradation
3	−0.1 ≤ ΔILC ≤ 0.1	Stable/ no change
4	0.1 < ΔILC ≤ 0.3	Moderate improvement
5	ΔILC > 0.3	Strong improvement

To assess changes in WUE across the five ILC categories, a spatial overlay analysis was performed using the zonal statistics tool in ArcGIS 10.2^70^. The ILC categorical map served as the zone layer, while annual WUE raster layers were used as input values. Mean WUE (g C mm^−1^) was calculated for each class for the baseline year (2000) and the final year (2024). Absolute and percentage changes in WUE were then computed for each ILC category. A Kruskal–Wallis test was performed to assess whether WUE differed significantly among the five ILC categories. Post hoc pairwise comparisons were conducted using Dunn's test with Bonferroni correction to identify which ILC category pairs differed significantly.

## Results

### LUCC detection

Based on the expanded independent validation dataset, the land use/land cover (LULC) classification achieved overall accuracies of 82.0% and 81.0%, with corresponding kappa coefficients of 0.80 and 0.78 for the 2000 and 2024 maps, respectively (Table 3). These values represent updated estimates of classification accuracy derived from a more comprehensive validation dataset. The revised assessment provides a more robust estimate of map accuracy by incorporating additional reference samples across all land-cover classes. The accuracy assessment confusion matrices can be found in the supplementary material (Table S1 & S2). Producer's and user's accuracies varied among LULC classes. Reed vegetation exhibited the lowest producer's accuracy in 2024 (0.51), while wetlands and urban areas showed relatively lower user's accuracies compared with other classes. These results indicate that commission and omission errors were concentrated in a limited number of spectrally similar classes, whereas the overall classification demonstrated considerable agreement with the reference data.

**Table 3:** Overall accuracy, kappa coefficient, producer’s and user’s accuracy for the 2000 and 2024 land use/land cover (LULC) maps.

LULC class	2000		2024	
	Producer’s accuracy	User’s accuracy	Producer’s accuracy	User’s accuracy
Urban	0.76	0.62	0.8	0.69
Wetlands	0.63	0.59	0.74	0.6
Agriculture	0.76	0.92	0.88	0.83
Reclaimed land	0.91	0.9	0.91	0.87
Sand plains	0.68	0.85	0.69	0.69
Salt marsh	0.98	0.96	0.93	0.98
Water	0.78	0.68	0.66	0.86
Reed vegetation	0.77	0.7	0.51	0.68
Bare rock	0.99	0.92	0.86	0.97
Gravel/Sand flats	0.87	0.88	0.81	0.84
% overall accuracy (OA)	82.0		81.0	
Kappa	0.80		0.78	

**Fig. 2:**
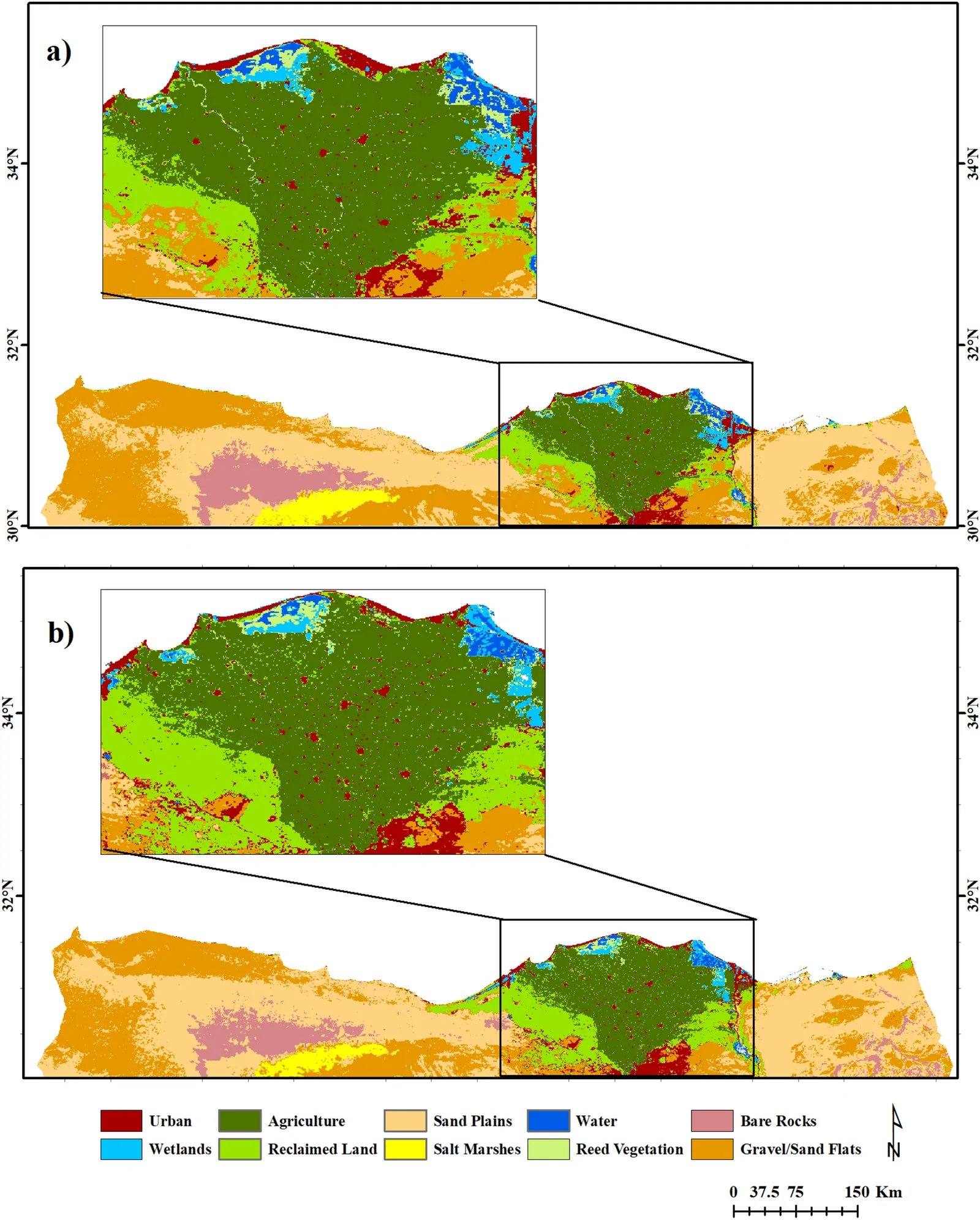
Land use/land cover (LULC) maps of the study area showing major LULC classes. (**a**) LULC map for 2000; (**b**) LULC map for 2024. The inset maps highlight an enlarged section of the Deltaic coastal area of Egypt. Map generated by authors using ArcMap 10.3^83^ https://www.esri.com.

**Fig. 3:**
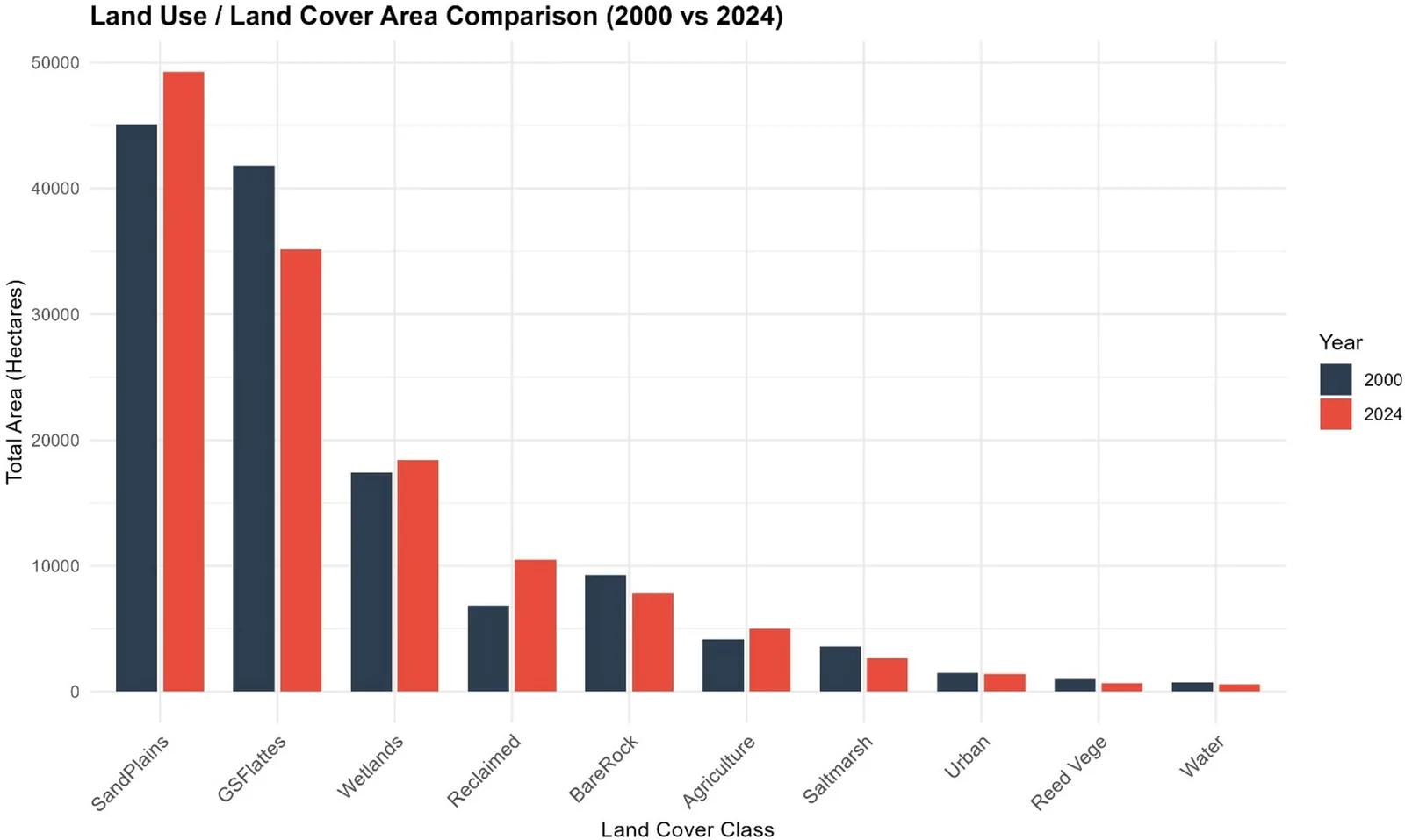
Total change in each land cover class over the study period (2000–2024). Values are represented in absolute area (hectares).

**Fig. 4:**
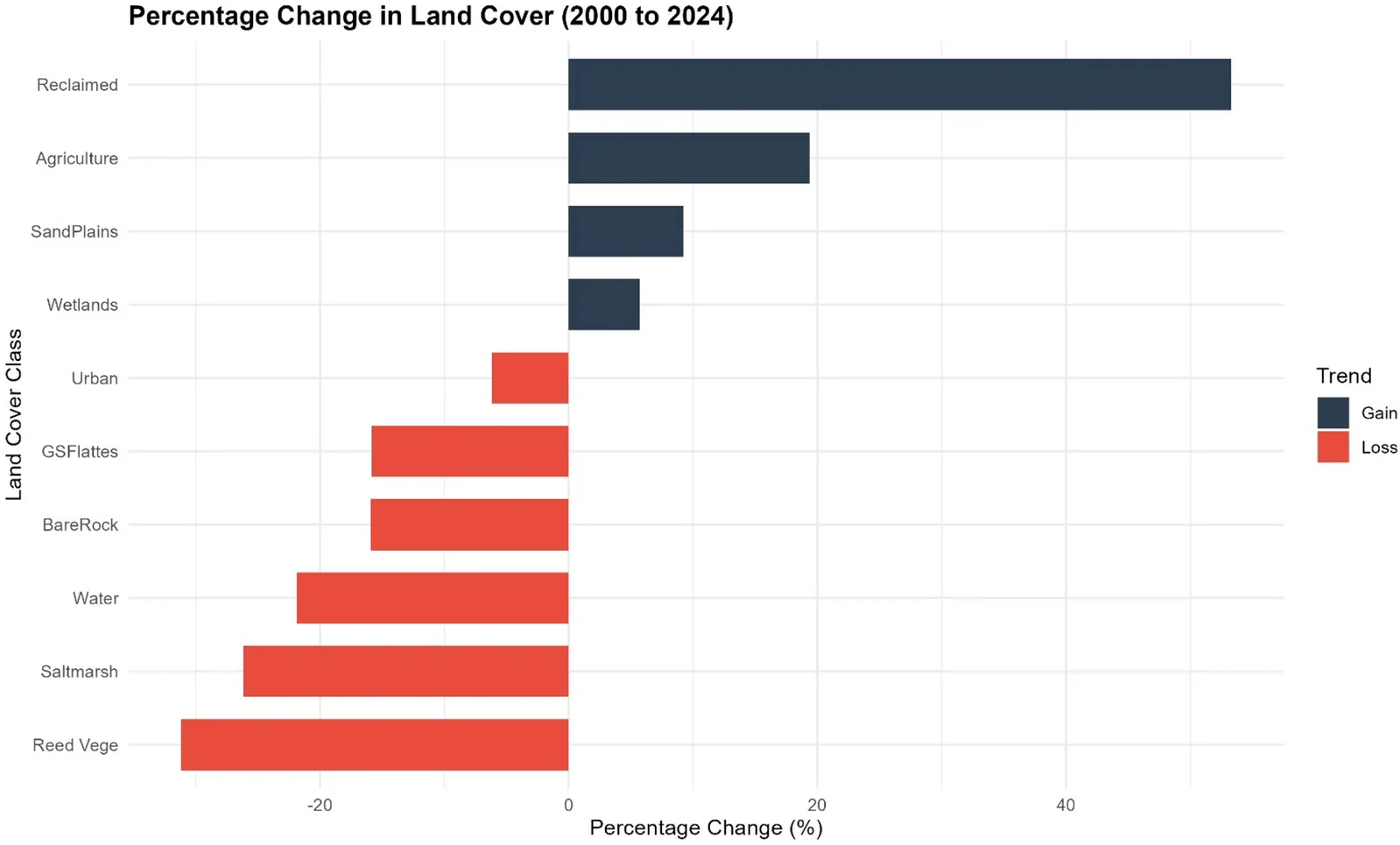
Percentage of change in each land cover over the period 2000–2024. Red indicates the losses and blue the gains.

The analysis of land cover change between 2000 and 2024 reveals notable transformations across different categories. The most notable change occurred in the reclaimed lands, which grew from 6,843 km^2^ to 10,490 km^2^ representing more than 53.3% increase in its area (Figs. 2 and 3). The agricultural area has increased from 4,177 km^2^ in 2000 to 4,987 km^2^ in 2024 (about 19.4% expansion). The wetlands also increased by 5.7%, which is nearly equal to 998 km^2^. The sand plains expanded from 45,101 km^2^ to 49,258 km^2^_,_ which is more than 9.2%. Overall, the largest absolute gain was in sand plains that gained more than 4,157 km^2^, followed by reclaimed land, gained more than 3,647 km^2^ (Fig. 4). The largest relative gain among LULC categories was attained by reclaimed land that gained more than 53% of its area.

### Spatiotemporal patterns & trends of water use efficiency (WUE)

**Fig. 5:**
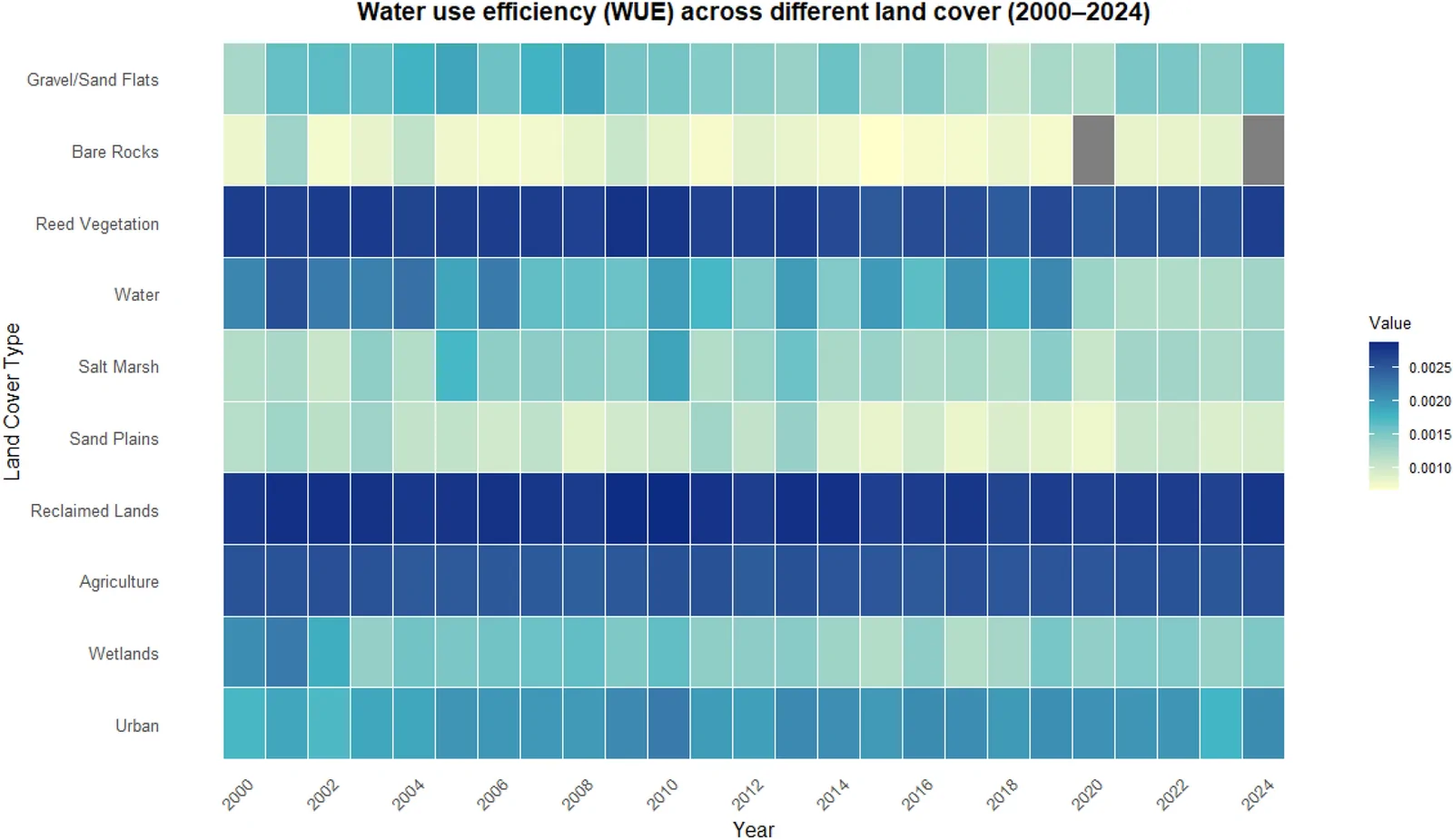
Heatmap of water use efficiency (WUE) across different land cover classes from 2000 to 2024.

**Fig. 6:**
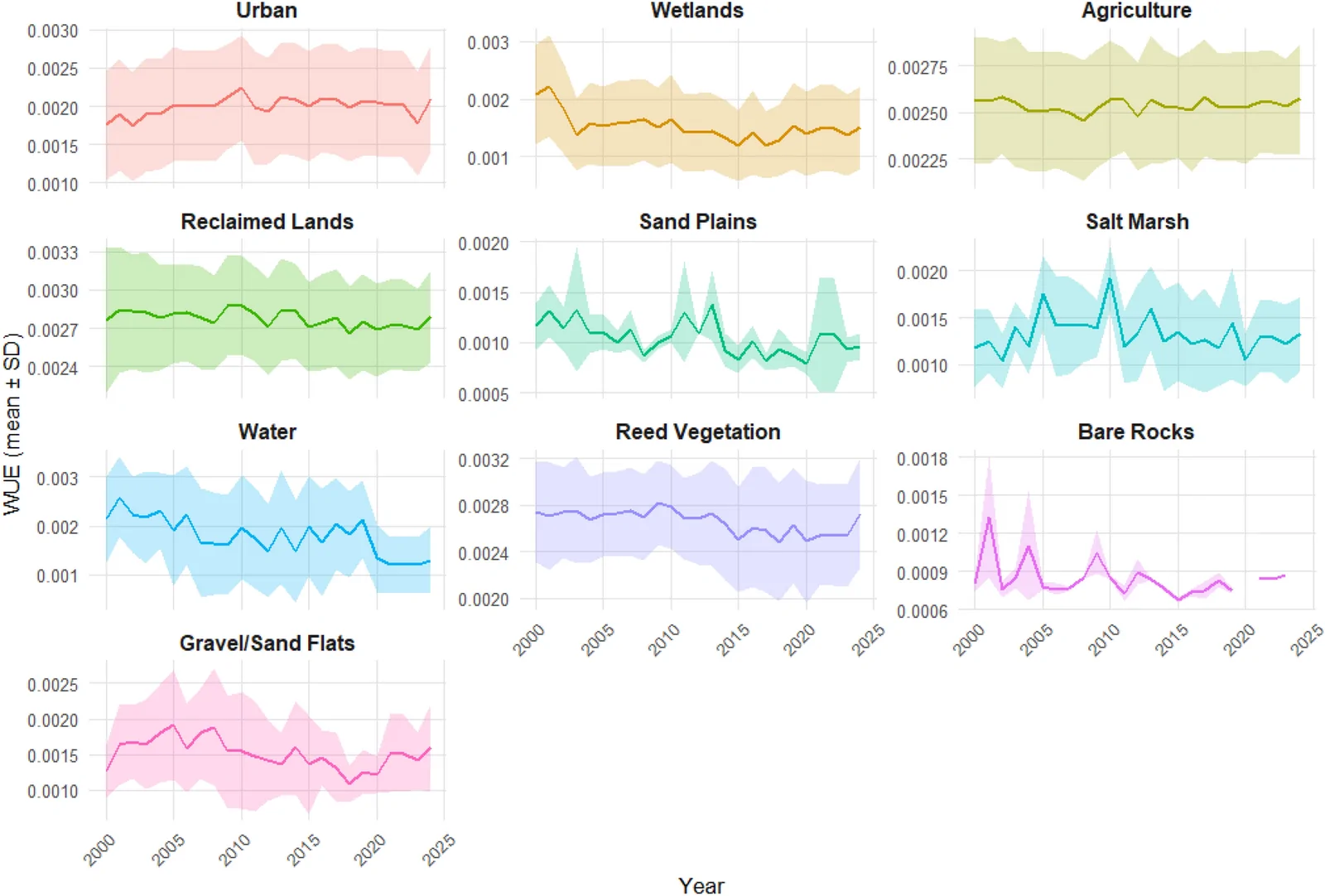
Temporal trends in water use efficiency (WUE) across different land cover classes in Egypt’s Mediterranean coastal zone from 2000 to 2024.

Heatmap of the WUE for the different LULCs across the study period (Fig. 5) indicates that the two land covers attaining the highest WUE values across time are the reclaimed lands and reed vegetation. The bare rocks and sand plains show the lowest values. While urban and wetlands show moderate fluctuations, their values remain relatively stable across time. Sand plain and salt marsh show more variability, with noticeable dips and peaks over different years (Fig. 6). The LULC category of gravel and sand flats tends to have consistent mid-range values over time.

The highest values that represent stable categories over time are reclaimed lands and reed vegetation (Fig. 6). Reclaimed lands show consistently the highest values ranging from 2.7 × 10^−3^ to 2.9 × 10^−3^ g-C mm^−1^ over the years while the Reed Vegetation are very stable and shows values around 2.6 × 10^−3^ g C mm^−1^. The agriculture areas have moderate value of 2.5 × 10^−3^ g C mm^−1^ with slightly downward trend after 2010. On the other side, salt marsh land cover has big oscillations ranging from 1.4 × 10^−3^ g C mm^−1^ to 2.2 × 10^−3^ g C mm^−1^ with no clear trend. The wetlands are slightly declining from 1.3 × 10^−3^ to 1.7 × 10^−3^ g C mm^−1^. The Gravel/Sand Flats are steady with mild ups and downs. The lowest values are indicated in sand plains and bare rocks with small fluctuations.

The Mann–Kendall test combined with Sen’s slope estimator results (Table 4) revealed divergent trends in WUE across the land cover classes, with the majority showing significant declining trends over time. Urban areas exhibit a small but statistically significant increase in WUE (Sen’s slope = 6.80 × 10^−6^, *p* = 0.045), suggesting a gradual improvement in water use efficiency over time. Strong and significant declines are observed in water bodies (−3.78 × 10^−5^, *p* < 0.001), reed vegetation (−9.24 × 10^−6^, *p* = 0.0011), sand plains (−1.16 × 10^−5^, *p* = 0.0038), wetlands (−1.47 × 10^−5^, *p* = 0.0047), gravel/sand flats (−1.67 × 10^−5^, *p* = 0.0077), and reclaimed lands (−5.00 × 10^−6^, *p* = 0.013). These results indicate a consistent decline in WUE across both natural and semi-natural ecosystems, with the most pronounced decrease occurring in water-related and wetland-associated classes. Agriculture, salt marsh, and bare rocks show no statistically significant trends (*p* > 0.05), indicating relatively stable WUE over the study period or variability without a clear directional pattern.

**Table 4:** Trend analysis of WUE values using the Mann–Kendall test and Sen’s slope estimator.

Land Cover	Sen's slope (g C $${\mathrm{m}\mathrm{m}}^{-1}$$$${\mathrm{y}\mathrm{r}}^{-1}$$)	Z-Score	*P*-value	Significance
Urban	6.80 × 10^−6^	2.01	0.045*	Significant
Wetlands	− 1.47 × 10^−5^	− 2.83	0.0047*	Significant
Agriculture	5.51 × 10^−7^	0.54	0.591	Not-significant
Reclaimed lands	− 5.00 × 10^−6^	− 2.48	0.013*	Significant
Sand plains	− 1.16 × 10^−5^	− 2.90	0.0038	Significant
Salt marsh	− 2.34 × 10^−6^	− 0.30	0.761	Not-significant
Water	− 3.78 × 10^−5^	− 3.50	< 0.001	Significant
Reed vegetation	− 9.24 × 10^−6^	− 3.27	0.0011	Significant
Bare rocks	− 4.84 × 10^−6^	− 1.61	0.107	Not-significant
Gravel/Sand flats	− 1.67 × 10^−5^	− 2.66	0.0077	Significant

**Fig. 7:**
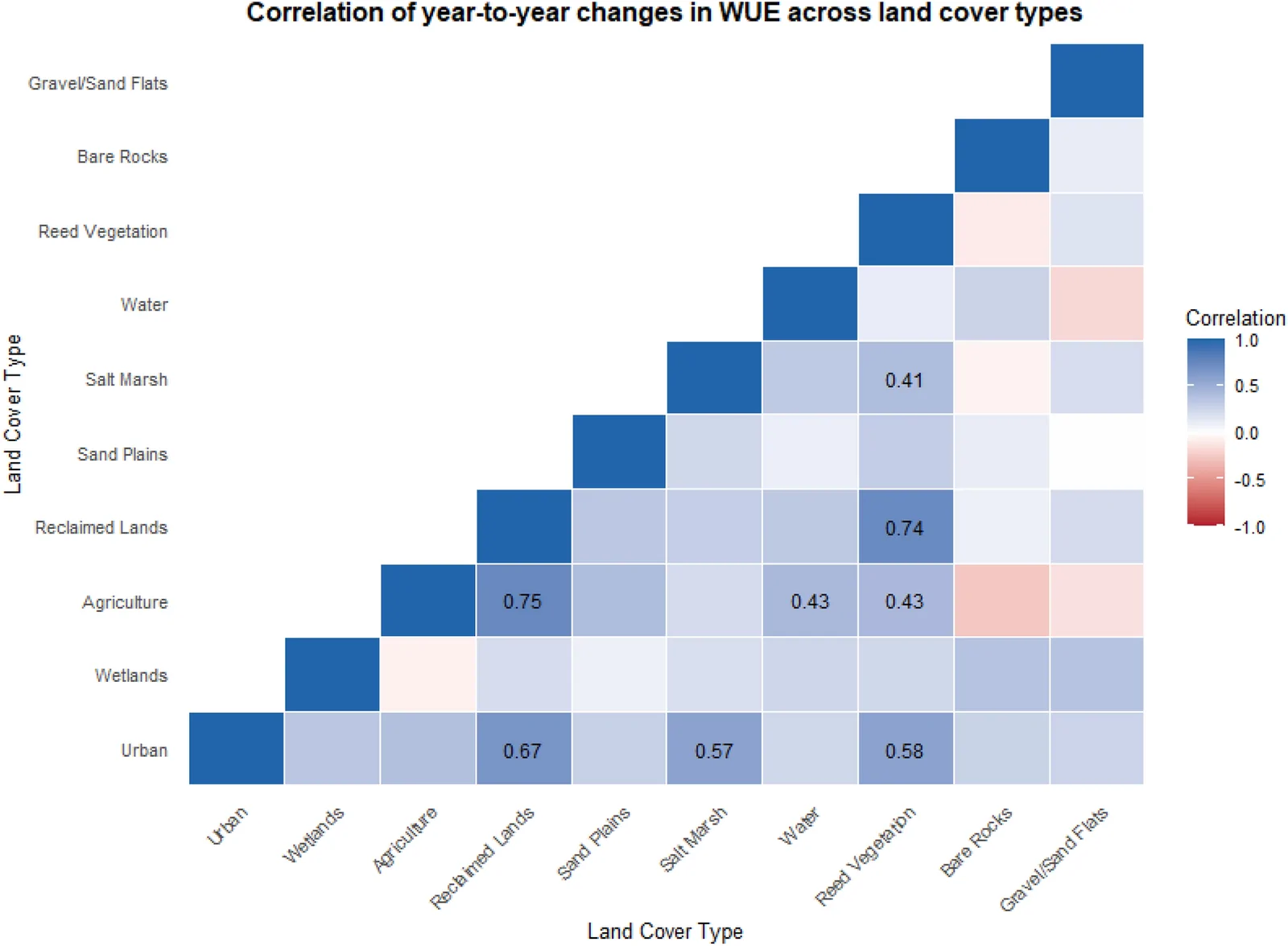
The correlogram matrix shows Pearson correlation coefficients for year-to-year changes in water use efficiency (WUE) among different land cover types. Positive (blue) and negative (red) correlations indicate the direction and strength of associations. Significant Pearson’s r correlation coefficient values are indicated within the cells of the matrix. Full Pearson correlation coefficients and their associated p-values are provided in Table S3 & S4 in the Supplementary Material.

The correlation matrix (Fig. 7; full results in Table S3 & S4, Supplementary Material) summarizes pairwise Pearson correlation coefficients (r) of interannual variations in WUE among land use/land cover (LULC) classes. Strong positive correlations are observed between agriculture and reclaimed lands (r = 0.75, *p* < 0.001), as well as between reclaimed lands and reed vegetation (r = 0.74, *p* < 0.001), and between urban areas and reclaimed lands (r = 0.67, p < 0.001). These associations indicate synchronous temporal variability in WUE among these classes, which may reflect shared external drivers or coincident responses to environmental conditions rather than direct interactions.

Moderate correlations are detected between salt marsh and reed vegetation (r = 0.41, *p* < 0.05), urban and salt marsh (r = 0.57, *p* < 0.01), urban and reed vegetation (r = 0.58, *p* < 0.01), and agriculture with both reed vegetation and water (r = 0.43, *p* < 0.05). However, correlations of lower magnitude (e.g., r < 0.4) are generally weak and, in some cases, not statistically significant, highlighting the importance of considering uncertainty in these estimates.

Overall, higher correlations are primarily observed among human-dominated land cover types (urban, reclaimed lands, and agriculture), suggesting similar temporal patterns in WUE variability. In contrast, sand plains, bare rocks, and gravel/sand flats exhibit weak or inconsistent correlations with other classes, indicating more independent temporal dynamics.

### Pattern of landscape conversion and implications for WUE

The map of ILC index provided a continuous representation of local landscape condition, ranging from (–1) fully man-made to (+ 1) fully natural. The man-made landscape is represented by agricultural cover, reclaimed land area, urban settlements, and areas undergoing intensive development near the coastal shorelines. The natural landscapes are represented by salt marshes, reed vegetation zones, and extensive sand plains and bare rocks.

**Fig. 8:**
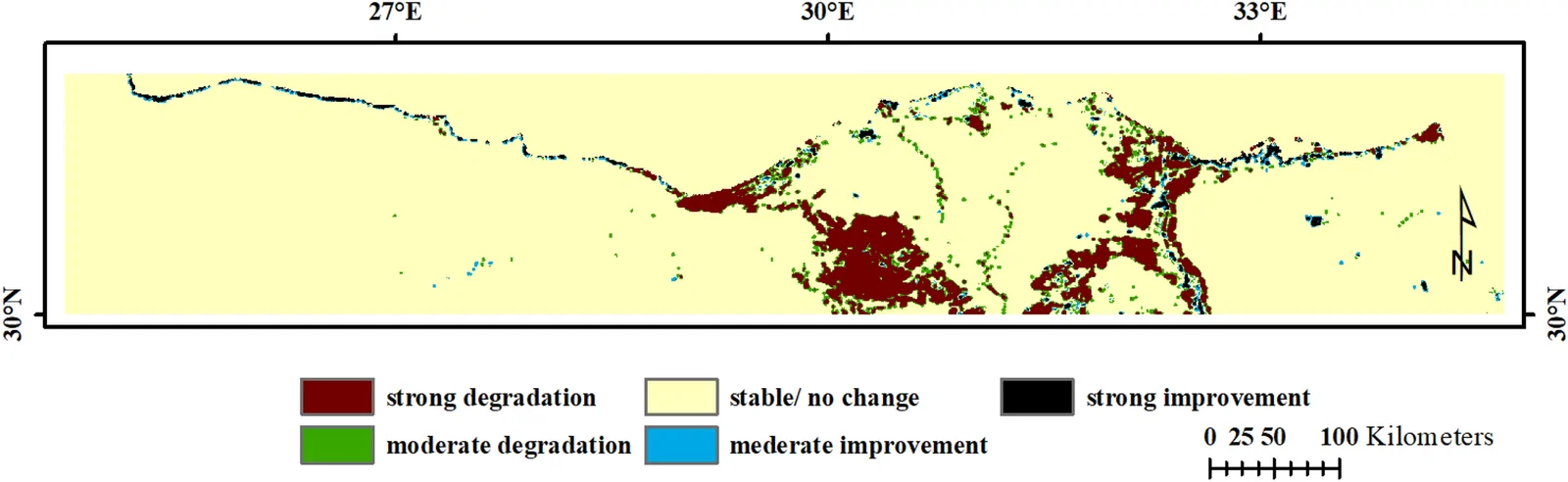
Pattern of landscape conversion and degradation as assessed by the landscape conservation index (ILC). Map generated by authors using ArcMap 10.3^83^ https://www.esri.com.

**Fig. 9:**
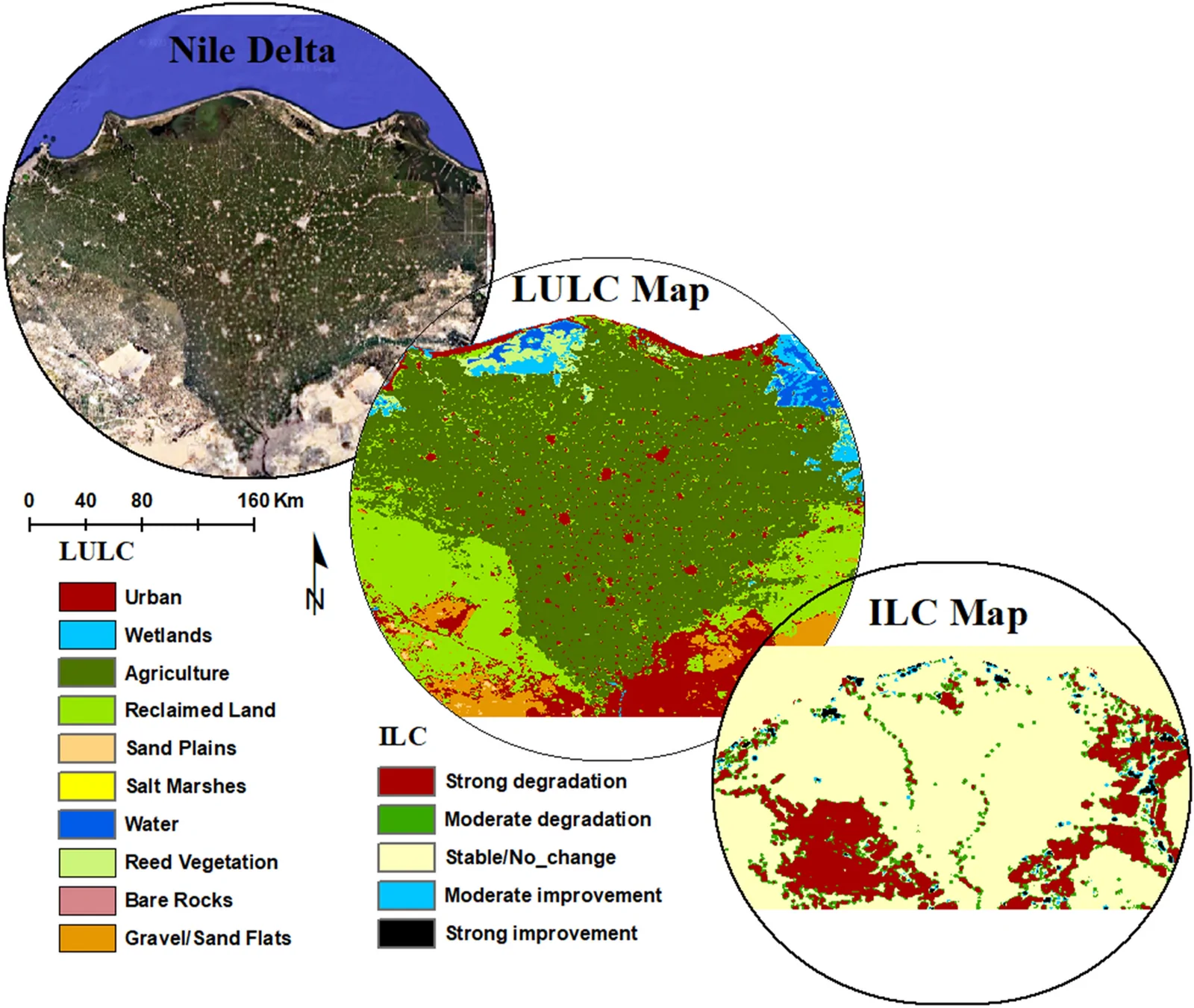
The LULC and ILC pattern in the Nile Delta. Map generated by authors using ArcMap 10.3^83^ https://www.esri.com .

The majority of the area falls into the stable class which exists in the vast sandy flats in the western desert area and in Sinai Peninsula which reflects little land-use pressure. This suggests that the habitat integrity was not heavily altered across the study area. Degradation is concentrated near areas of infrastructure expansion, particularly through the growth of urban settlements and reclaimed lands, reflecting changes in natural land cover. Those areas classes can be noticed east and west of the Nile delta as represented in Figs. 8 and 9. As well as areas in the coastal shoreline that reflects the coastal developments. Very small regions of recovery exist within the area and represent only 1.75% of the total study area.

The analysis of WUE across different ILC classes from 2000 to 2024 reveals distinct regional trajectories based on conversion status (Table S5, Supplementary Material). An increase in WUE was noticed across all categories. The Stable category, representing the traditional agricultural core of the Delta, maintained the highest absolute WUE in both 2000 (0.00259) and 2024 (0.00262), but exhibited near-stagnant relative growth (1.07%). The most significant dynamic occurred in the Strong degradation category which corresponds geographically to the eastern and western desert fringes undergoing active land reclamation. In these areas, WUE experienced a striking 22.5% increase. On the other side, the lands showing Moderate and Strong improvement experienced steady WUE gains of 10.7% and 11.4% respectively and their baseline absolute WUE remained consistently lower than that of degraded or stable lands. The Kruskal–Wallis test (Table S6, Supplementary Material) indicated significant differences in WUE among the five ILC categories (χ^2^ = 3625.2, df = 4, *p* < 0.001). Post hoc Dunn's tests with Bonferroni correction showed significant differences between all category pairs except moderate and strong improvement.

## Discussion

### Trend of WUE

In northern Egypt, particularly along the Nile Delta fringes and the Mediterranean coastal desert, carbon–water exchange is closely linked to the region’s semi-arid climate and fragile ecosystems. Water scarcity driven by low rainfall and increasing evapotranspiration strongly limits vegetation cover and soil carbon storage^84^. In arid and semi-arid ecosystems, limited precipitation constrains both photosynthesis and evapotranspiration, making water availability the primary control on carbon fluxes^85^. These drylands are highly sensitive to climate change, rising temperatures and altered precipitation patterns reduce water-use efficiency (WUE) and substantially increase ecosystem vulnerability^86^.

Advances in agricultural water management and modern technologies can substantially reduce water consumption and enhance WUE in arid and semi-arid regions (Wang^14^,). In 2016, Egypt’s water deficit was estimated at 13.5 billion cubic meters per year, and it was projected to reach 26 billion cubic meters per year by 2025^87^. In coastal wetlands and delta ecosystems, irrigation and groundwater inputs help sustain productivity; however, salinization and reduced Nile discharge increasingly disrupt the balance between carbon uptake and water cycling^88^.

Globally, WUE varies across spatial scales and among ecosystems, reflecting differences in climate, hydrology, and vegetation structure^14^. The performance of MODIS dataset as reliable measures for WUE estimation was evaluated by different studies. Some studies indicated good relationship through validation of MODIS WUE with on-site flux data measurements^26,89^. While some studies refer to inaccurate results due to underestimation of MODIS GPP and overestimation of MODIS ET^90–92^. Spatial patterns of annual WUE derived from MODIS datasets may exhibit anomalous values in subtropical arid and semi-arid regions^34,91^. Previous studies have also reported that MODIS-based evapotranspiration (ET) tends to be underestimated in such environments^91,93,94^. Nevertheless, comparisons with independent datasets suggest that MODIS products are capable of capturing ecosystem disturbances and broader global trends in WUE^89^.

In the absence of in situ measurements within the study area, we evaluated the reliability of our results by comparing the estimated WUE values with ranges reported in the literature for similar ecosystems. As shown in Table 5, the WUE values obtained in this study fall within the reference ranges for the corresponding land cover classes, supporting the reliability of our estimates. Noticeably, the reclaimed lands are slightly above the reference range with a mean WUE value of 2.78.

**Table 5:** Comparison of WUE values measured in this study with ranges reported in previous studies.

Land cover class	Mean WUE (g C ${\mathrm{m}\mathrm{m}}^{-1}$)	Reference range (g C ${\mathrm{m}\mathrm{m}}^{-1}$)	Key references
Reclaimed lands	2.78	0.6 – 2.7	Zwart & Bastiaanssen^95^
Reed vegetation	2.66	1.28 – 3.01	Zhang et al.^96^ Liu et al.^97^
Agriculture	2.54	0.92 – 3.16	Zhu et al.^98^
Wetlands	1.52	0.57 – 3.01	Zhu et al.^98^
Salt Marsh	1.34	1.0 – 1.63	Tang et al.^26^ Zhu et al.^98^

In the present study WUE was evaluated in arid ecosystems existing in the north area of Egypt. Overall, WUE values showed slight variation over time but remain relatively stable over the 25-year period. The highest WUE, observed in 2010, suggests that reduced evapotranspiration (ET) may have resulted from relatively lower temperatures or drought conditions during that year. The study of Xue et al.^89^, indicates that in 2010 drought affected large areas of Europe showing a significant decrease in WUE value in the European region. Under drought conditions, and when the vegetation is relatively sparse, evaporation from bare soil tends to increase substantially affecting the capacity of plant for producing organic matter. Drought conditions raise atmospheric evaporative demand; therefore, ecosystems may exhibit a pronounced reduction in water use efficiency (WUE) when subjected to extreme drought stress^99^. Drought directly disrupts vegetation transpiration and soil evaporation, which in turn impacts photosynthesis and the ecosystem WUE of vegetation^7,100^. The assessment of the trends in WUE across the different land cover types revealed contrasting trends in WUE across land cover types, with the majority showing significant declining trends over time. Overall, the results suggest a general decline in WUE across most natural land cover types, particularly those associated with water and wetland systems, while urban areas show a slight increasing trend. However, although statistically significant, the magnitude of Sen’s slopes is relatively small, indicating gradual changes over time. These findings should be interpreted as temporal trends rather than causal relationships, and potential drivers (e.g., climate variability, hydrological changes, or land management practices) require further investigation.

### Impact of land use change on WUE

The land cover classes existing in the study area can be categorized into three levels: Human-driven processes, Natural/fragile ecosystems, and Low-value classes. The Human-driven processes are Urban, Agriculture, and Reclaimed Lands tend to correlate positively with each other. These landcover changes tend to move together, reflecting urban and reclamation projects expansion and associated agricultural development. In the last 50 years, numerous government-led desert reclamation projects have been carried out, leading to the conversion of more than 12,000 km^2^ of desert into farmland^101^. Estimation of 252 km^2^ of Egypt’s fertile cropland is lost annually due to expanding of urban landscapes^101^.

Urbanization and reclamation are tightly bound processes, indicating the ongoing changes in the study area. The reclamation activities increased by more than 40% reflecting the development projects initiated west of the Nile delta. The expansion in these agricultural lands changes in the water consumption in the area. These development projects are mainly dependent on modern irrigation techniques. The technology of irrigation applied in most of the fields is a mixture of center-pivot irrigation and drip irrigation using central irrigation machines fed by groundwater pumps^102^. In cropland ecosystems, the water management techniques such as drip irrigation can significantly reduce evaporation thus reduced ET and increase WUE^14,103^.

The western desert reclamation areas are characterized by high air and soil temperatures, elevated evaporation rates, strong winds, and low relative humidity^104,105^. These conditions are expected to result in relatively low WUE. However, our data suggests that local agricultural practices may exert a stronger influence on WUE than previously recognized, potentially providing new opportunities for improving sustainable water use. Conversely, urbanization tends to increase evapotranspiration and reduce WUE, as demonstrated by Tang et al.^7^. Similarly, Fu et al.^35^ reported that the expansion of artificial and impervious surfaces can lead to declines in the WUE of surrounding natural ecosystems.

The natural/fragile ecosystems are wetlands, salt marsh, and open water which show mixed or weak correlations, suggesting more complex dynamics. Those land covers show more independent or opposing trends, likely to reflect environmental variability rather than coordinated land management. Salt marshes are changing naturally over the years, and the wetlands are changing according to the surrounding development planning. Studies indicated that Mediterranean wetlands are being converted into aquaculture farms to meet the needs of the growing population and for their economic benefits^101,106,107^. Other studies indicated that the release of agricultural and municipal wastes into wetlands ecosystems without proper treatment promotes the growth of emergent grasses and reeds, resulting in an expansion of reed-covered areas^107–109^. Therefore, the dynamic changes happen in these natural ecosystems is related to nearby development and land use strategies.

The sand plains, bare rocks, and gravel/sand flats LULC classes are weakly correlated overall, sometimes even negatively with agriculture or water. Halmy^65^ and El-Masry et al.^110^ attributed the decrease in the bare land area in the coastal area of the western Mediterranean coast to the increase in the built-up areas and the uncontrolled mining of limestone and other activities like road constructions. Different other studies reported the land use change in the Mediterranean western coast of Egypt^41,63,64,111,112^. These remarkable changes are due to the development projects such as tourism, agriculture or expansion of urbanized and industrial areas (e.g., Burg El Arab Region).

### Landscape conservation, WUE, and implications for land use planning

The index of landscape conservation (ILC) provides a decision-support tool for identifying pressure hotspots, prioritizing conservation zones, informing sustainable land-use planning, and supporting coastal management strategies^81,82^. Areas with declining ILC should be monitored to prevent further ecosystem degradation, especially where ecosystems are characterized with high economic values and near sensitive wetlands. The ILC map provides an important spatial layer that helps interpret ecosystem functional patterns. The strong degraded classes are noticeable east and west of the Nile delta, where the wide expansion in urban and development activities dominates the land use changes reflecting intensification of human activities and loss of semi natural habitat. The coastal habitat is affected by strong and moderate degradation where the primary causes of land cover modifications are summer resort development and irrigation system construction. In some areas, such as El-Omayed Biosphere Reserve (OBR), these activities have caused the loss of nearly 83% of the coastal dunes and have led to the degradation of the natural habitat of non-saline depressions^113^.

The observed rise in WUE within the Strong degradation class initially appears contradictory, but it reflect the extensive agricultural reclamation activities occurring on the eastern and western desert fringes of the Nile Delta. In these zones, the conversion of barren arid land to intensive agriculture drives a rapid spike in Gross Primary Production (GPP). Because these new reclamations rely heavily on pressurized, modern irrigation systems, such as drip and pivot sprinklers^102^, evaporative water loss is strictly minimized. Consequently, the ratio of carbon assimilation to water loss is mathematically rises. However, the classification of these lands as strongly degraded reflects the hidden ecological costs of this localized efficiency which might include severe soil salinization, the rapid drawdown of fossil groundwater aquifers, and the complete displacement of native desert ecosystems^114,115^.

On the contrary, the traditional agricultural core of the Delta, categorized as Stable, exhibits a biological and agronomic ceiling. While the nutrient-rich alluvial soils sustained the highest absolute WUE across the study period, the relative growth is effectively stagnant. This stagnation is a dual consequence of historically maximized crop yields (a GPP ceiling) and the persistent reliance on traditional flood irrigation, which maintains continuously high Evapotranspiration (ET). The system is highly productive but has exhausted its capacity for rapid efficiency gains without sweeping infrastructural modernization^116^.

The relationship between moderate and strong improvement areas’ absolute WUE highlights a fundamental divergence between conservation and agricultural metrics. Lands showing ecological recovery demonstrate steady relative WUE growth but maintain the lowest absolute baselines. Natural wetland ecosystems and native flora naturally incur high ET rates without the artificially inflated carbon assimilation characteristic of heavily fertilized, commercial agriculture.

The Kruskal–Wallis test confirmed that WUE differed significantly among ILC categories (*p* < 0.001). In this context, the ILC framework serves as a valuable structural indicator for interpreting ecosystem functioning. Conversely, highly transformed landscapes, such as urban settlements, newly reclaimed lands, and coastal shoreline developments, formed distinct spatial clusters corresponding to zones of concentrated human activity, further highlighting the strong imprint of anthropogenic processes on landscape structure and function.

### Limitations and future directions

Although the overall classification accuracy (81–82%) and Kappa coefficients (0.78–0.80) indicate considerable agreement between the classified maps and the reference data^117^, uncertainty remains for several individual LULC categories. In particular, reed vegetation, wetlands, and urban areas exhibited comparatively lower producer's or user's accuracies than the remaining categories. These errors are primarily attributable to the moderate spatial resolution (500 m) of the MODIS data, the relatively large number of LULC categories considered in the study, and the heterogeneous nature of the study landscape. Under these conditions, mixed pixels and spectral similarity between adjacent categories, particularly agricultural land, reclaimed land, and reed vegetation, can increase commission and omission errors, a well-recognized limitation of coarse-resolution land cover products^118,119^.

The relatively lower accuracy of reed vegetation and wetlands introduces additional uncertainty into the corresponding WUE estimates for these classes. Therefore, the reported WUE values and temporal trends should be interpreted as representative of large-scale patterns rather than precise estimates at the individual-pixel level. Nevertheless, because both the land cover classification and the MODIS GPP and ET products used to calculate WUE are derived at comparable spatial resolutions, the analysis remains appropriate for assessing broad spatial and temporal variations in ecosystem water-use efficiency across the study area.

Direct measurements of water and carbon fluxes remain limited in Egypt, primarily due to gaps in monitoring infrastructure, which constrain the availability of site-level observations and restrict the use of direct field measurements in this study. Consequently, the analysis relies on remotely sensed datasets, which are valuable for characterizing broad spatial patterns of water use and carbon assimilation across arid ecosystems, inherently introduce uncertainty. In particular, the coarse spatial resolution of MODIS data (500 m) represents a key limitation for land use/land cover (LULC) classification and the subsequent assessment of water use efficiency (WUE) across LULC categories. At this resolution, individual pixels frequently contain multiple land cover types, especially in heterogeneous or fragmented landscapes, leading to mixed-pixel effects and potential misclassification. These uncertainties can propagate into WUE estimates by integrating evapotranspiration and carbon flux signals from adjacent or co-occurring land-cover types, thereby reducing the precision of LULC-specific WUE values^120–122^. As a result, the reported WUE patterns should be interpreted as indicative of dominant land-cover characteristics at regional scales rather than fine-scale ecosystem processes. Additionally, observed correlations among WUE time series should be understood as indicators of temporal co-variation rather than evidence of causal interactions between land cover types. Therefore, these relationships do not directly reflect land cover transitions or conversion processes and should be interpreted with appropriate caution. The gap in flux measurements across Egyptian landscapes highlights the need to establish dedicated monitoring infrastructure (e.g., eddy-covariance towers) to strengthen national observation capacity and facilitate Egypt’s integration into the global flux monitoring network.

## Conclusion

Dryland vegetation in northern Egypt, including sparse shrublands and agricultural fields, is strongly constrained by water availability, which in turn limits photosynthetic activity and carbon sequestration potential. The region comprises a mosaic of natural and human-modified land covers. Natural ecosystems are fragile and continue to experience change due to human intervention, whereas wetlands, salt marshes, and open-water bodies are closely linked to nearby development and land-reclamation activities. Extensive sand plains and flats have also undergone rapid transformation, particularly along the coastal strip, due to expanding built-up areas.

The highest values of WUE were observed in vegetated areas such as croplands and reed communities, where biomass reflects actively growing vegetation. However, high temperatures and elevated evaporation rates increase water loss, ultimately reducing effective WUE. Notably, the highest WUE recorded in 2010 likely reflects reduced evapotranspiration associated with relatively lower temperatures or drought conditions during that year.

Because this study focuses specifically on northern Egypt, results should be extrapolated cautiously to broader regional contexts. Nevertheless, the findings provide important insights into the relationships among agricultural practices, environmental conditions, and water resource management in dryland systems. While the study advances understanding of WUE patterns, certain constraints remain, particularly related to data availability. Future research should expand spatial and temporal coverage and place greater emphasis on the interactions between ecosystem conditions, WUE, and carbon assimilation, including the role of biological processes^13,122–125^.

## Supplementary Information

Supplementary Information.

## Data Availability

The raw MODIS MOD16A2GF and auxiliary datasets used in this study are publicly available through the NASA Earthdata Search and the Google Earth Engine (GEE) Data Catalog. The GEE JavaScript scripts developed for classification are available at [GitHub Repository], the link for that is also provided in the Supplementary Material. Researchers can replicate the study's workflow using this script.
