# Supplementary material for: Assessment of water use efficiency in arid ecosystems of the coastal land of Egypt using MODIS dataset
Source: Sci Rep. 2026 Sep 5;16:27816. doi: 10.1038/s41598-026-67232-3 (PMC13546289; doi:10.1038/s41598-026-67232-3)
Supplement: Supplementary file 1 [file 41598_2026_67232_MOESM1_ESM.docx]

**Assessment of Water Use Efficiency in Arid Ecosystems of the Coastal Land of Egypt Using MODIS dataset**

**Amira M. Hotaiba^1a*^, Boshra B. Salem^1b^, Marwa Waseem A. Halmy^1c^**

**1 Department of Environmental Sciences, Faculty of Science, Alexandria University, Moharm Bek, P.O. Box: 21511, Alexandria, Egypt**

**^a^** [**amira.hotaiba@alexu.edu.eg**](mailto:amira.hotaiba@alexu.edu.eg) **, ORCID:** [**https://orcid.org/0000-0001-5305-1555**](https://orcid.org/0000-0001-5305-1555)

**^b^** [**Boshra.salem@alexu.edu.eg**](mailto:Boshra.salem@alexu.edu.eg) **, ORCID:** [**https://orcid.org/0000-0001-9715-6324**](https://orcid.org/0000-0001-9715-6324)

**^c^** [**Marwa.w.halmy@alexu.edu.eg**](mailto:Marwa.w.halmy@alexu.edu.eg) **, ORCID:** [**https://orcid.org/0000-0002-4183-973X**](https://orcid.org/0000-0002-4183-973X)

**Supplementary Table S1.** Confusion matrix for 2000 LULC map

| **LULC Categories** | **Urban** | **Wetlands** | **Agriculture** | **Reclaimed Land** | **Sand Plains** | **Salt Marsh** | **Water** | **Reed Vegetation** | **Bare Rock** | **Gravel & Sand Flats** |
| --- | --- | --- | --- | --- | --- | --- | --- | --- | --- | --- |
| **Urban** | 97 | 3 | 15 | 9 | 1 | 0 | 0 | 1 | 1 | 1 |
| **Wetlands** | 7 | 61 | 0 | 0 | 0 | 0 | 23 | 6 | 0 | 0 |
| **Agriculture** | 18 | 29 | 198 | 4 | 0 | 0 | 0 | 11 | 0 | 0 |
| **Reclaimed Land** | 5 | 0 | 1 | 180 | 0 | 0 | 0 | 0 | 0 | 11 |
| **Sand Plains** | 23 | 2 | 0 | 4 | 102 | 3 | 2 | 0 | 10 | 5 |
| **Salt Marsh** | 3 | 0 | 0 | 0 | 0 | 143 | 0 | 0 | 0 | 0 |
| **Water** | 3 | 8 | 1 | 1 | 1 | 0 | 91 | 8 | 0 | 0 |
| **Reed**  **Vegetation** | 0 | 0 | 0 | 0 | 0 | 0 | 18 | 61 | 0 | 0 |
| **Bare Rock** | 0 | 0 | 0 | 0 | 1 | 0 | 0 | 0 | 128 | 0 |
| **Gravel & Sand Flats** | 0 | 0 | 0 | 1 | 15 | 3 | 0 | 0 | 0 | 128 |

**Supplementary Table S2.** Confusion matrix for 2024 LULC map.

| **LULC Categories** | **Urban** | **Wetlands** | **Agriculture** | **Reclaimed Land** | **Sand Plains** | **Salt Marsh** | **Water** | **Reed Vegetation** | **Bare Rock** | **Gravel & Sand Flats** |
| --- | --- | --- | --- | --- | --- | --- | --- | --- | --- | --- |
| **Urban** | 60 | 1 | 22 | 12 | 0 | 0 | 0 | 4 | 0 | 0 |
| **Wetlands** | 6 | 51 | 10 | 0 | 0 | 1 | 2 | 11 | 0 | 0 |
| **Agri-culture** | 14 | 19 | 191 | 19 | 0 | 0 | 0 | 17 | 0 | 0 |
| **Reclaimed Land** | 4 | 0 | 14 | 117 | 0 | 0 | 0 | 0 | 0 | 3 |
| **Sand Plains** | 45 | 5 | 0 | 10 | 126 | 4 | 0 | 0 | 4 | 17 |
| **Salt Marsh** | 2 | 0 | 0 | 2 | 3 | 125 | 0 | 0 | 0 | 8 |
| **Water** | 12 | 50 | 12 | 1 | 1 | 1 | 95 | 8 | 0 | 0 |
| **Reed**  **Vegetation** | 2 | 24 | 10 | 0 | 0 | 0 | 16 | 38 | 0 | 0 |
| **Bare Rock** | 0 | 0 | 0 | 0 | 27 | 0 | 0 | 0 | 115 | 0 |
| **Gravel & Sand Flats** | 6 | 0 | 0 | 1 | 39 | 2 | 0 | 0 | 0 | 100 |

**Supplementary Table S3.** Pearson correlation coefficients (r) of year-to-year WUE changes across land cover classes. Significance levels are indicated as ^*^p-value < 0.05, ^**^p-value < 0.01, ^***^p-value < 0.001.

| **LULC** | **Urban** | **Wetlands** | **Agriculture** | **Reclaimed Lands** | **Sand Plains** | **Salt Marsh** | **Water** | **Reed Vegetation** | **Bare Rocks** | **Gravel/ Sand Flats** |
| --- | --- | --- | --- | --- | --- | --- | --- | --- | --- | --- |
| **Urban** | 1 |  |  |  |  |  |  |  |  |  |
| **Wetlands** | 0.345 | 1 |  |  |  |  |  |  |  |  |
| **Agriculture** | 0.393 | -0.067 | 1 |  |  |  |  |  |  |  |
| **Reclaimed Lands** | 0.672^***^ | 0.215 | 0.755^***^ | 1 |  |  |  |  |  |  |
| **Sand Plains** | 0.291 | 0.088 | 0.400 | 0.340 | 1 |  |  |  |  |  |
| **Salt Marsh** | 0.575^**^ | 0.200 | 0.223 | 0.301 | 0.243 | 1 |  |  |  |  |
| **Water** | 0.243 | 0.249 | 0.434^*^ | 0.335 | 0.102 | 0.329 | 1 |  |  |  |
| **Reed Vegetation** | 0.578^**^ | 0.239 | 0.433^*^ | 0.741^***^ | 0.304 | 0.407^*^ | 0.112 | 1 |  |  |
| **Bare Rocks** | 0.272 | 0.369 | -0.258 | 0.093 | 0.117 | -0.054 | 0.267 | -0.099 | 1 |  |
| **Gravel/Sand Flats** | 0.268 | 0.369 | -0.138 | 0.22 | 0.008 | 0.205 | -0.179 | 0.173 | 0.118 | 1 |

**Supplementary Table S4.** P-value for the Pearson correlation coefficients (r) of year-to-year WUE changes across land use/cover classes (LULC).

| **LULC** | **Urban** | **Wetlands** | **Agriculture** | **Reclaimed Lands** | **Sand Plains** | **Salt Marsh** | **Water** | **Reed Vegetation** | **Bare Rocks** |
| --- | --- | --- | --- | --- | --- | --- | --- | --- | --- |
| **Wetlands** | 0.0989 |  |  |  |  |  |  |  |  |
| **Agriculture** | 0.0576 | 0.7557 |  |  |  |  |  |  |  |
| **Reclaimed Lands** | 0.0003^***^ | 0.3119 | 0.0000^***^ |  |  |  |  |  |  |
| **Sand Plains** | 0.1678 | 0.6811 | 0.0528 | 0.1040 |  |  |  |  |  |
| **Salt Marsh** | 0.0033^**^ | 0.3487 | 0.2955 | 0.1530 | 0.2529 |  |  |  |  |
| **Water** | 0.2527 | 0.2404 | 0.0340^*^ | 0.1100 | 0.6362 | 0.1164 |  |  |  |
| **Reed Vegetation** | 0.0031^**^ | 0.2601 | 0.0344^*^ | 0.0000^***^ | 0.1484 | 0.0483^*^ | 0.6018 |  |  |
| **Bare Rocks** | 0.2326 | 0.0997 | 0.2580 | 0.6880 | 0.6141 | 0.8155 | 0.2424 | 0.6709 |  |
| **Gravel/Sand Flats** | 0.2049 | 0.0758 | 0.5196 | 0.3011 | 0.9722 | 0.3372 | 0.4023 | 0.4196 | 0.6116 |

**Supplementary Table S5:** The absolute and relative changes of WUE for each ILC land conservation class for 2000 and 2024

| **ILC Class** | **Description** | **WUE (2000)** | **WUE (2024)** | **Absolute Change** | **Percentage Change** |
| --- | --- | --- | --- | --- | --- |
| 1 | Strong degradation | 0.00205 | 0.00251 | 0.000461 | 22.5% |
| 2 | Moderate degradation | 0.00247 | 0.00259 | 0.000122 | 4.97% |
| 3 | Stable | 0.00259 | 0.00262 | 0.0000278 | 1.07% |
| 4 | Moderate improvement | 0.00201 | 0.00222 | 0.000214 | 10.7% |
| 5 | Strong improvement | 0.00163 | 0.00181 | 0.000185 | 11.4% |

**Supplementary Table S6:** Kruskal-Wallis rank sum test for significance of difference in WUE among land transformation categories and Dunn's Pairwise Comparison of change in WUE between land transformation categories

```
Kruskal-Wallis rank sum test

data: x and g
Kruskal-Wallis chi-squared = 3625.1818, df = 4, p-value = 0
                      Dunn's Pairwise Comparison of x by g
                                  (Bonferroni)

Col Mean-│
Row Mean │          1          2          3          4
─────────┼────────────────────────────────────────────
       2 │   28.88973
         │     0.0000*
         │
       3 │   57.02020   14.46282
         │     0.0000*    0.0000*
         │
       4 │   7.016115  -7.501295  -13.92569
         │     0.0000*    0.0000*    0.0000*
         │
       5 │   8.949189  -5.970633  -12.48829   1.298472
         │     0.0000*    0.0000*    0.0000*    1.0000

FWER = 0.05
Reject Ho if adjusted p ≤ FWER, where (unadjusted) p = Pr(|Z| ≥ |z|)
Dunn (1964) Kruskal-Wallis multiple comparison
p-values adjusted with the Bonferroni method.
```

**Links for the codes used in this study:**

Hotaiba, A. A. M., Salem, B.& Halmy, M. (2026). Assessment of Water Use Efficiency (WUE) in Arid Ecosystems of the Coastal Land of Egypt Using MODIS Dataset. Zenodo. <https://doi.org/10.5281/zenodo.21284597>
